# Improvement upon the Adhesive-Strap Counter-Extension

**Published:** 1862-01

**Authors:** E. Andrews

**Affiliations:** Chicago


### Improvement Upon the Adhesive-strap Counter-extension.

TO THE EDITORS OF THE CHICAGO MEDICAL EXAMINER.

Gentlemen:—In the last number of the Examiner I gave an account of a new method of counter-extension by means of adhesive-straps in cases of fractured thigh. In that communication, I advised the use of three broad straps of a yard and a half in length; one of them was to be applied along the abdomen and chest, on the afflicted side and parallel to the axis of the body, thence loosely over the shoulder and applied down the back as far as the brim of the pelvis. The second strap was to commence on the sound side between the crest of the ilium and trochanter major, and pass obliquely upward and across the body, over the same shoulder as the previous strap, then diagonally down the back until it returned to the starting point, and crossed the other end above the trochanter. The third strap was to be applied around the waist in the manner of a belt. This dressing was used in the following manner: To the top of a Dessault's, or any other long splint, a bent iron was attached, curving around the bulge of the front of the shoulder, and terminating in a proper hook which was attached to the loose loop of the adhesive-straps where they crossed the top of the shoulder. Adhesive-straps were also used in the ordinary way for the extension. All surgeons know the perfect comfort and ease of the adhesive-strap extension, and the pain, vexation and distress of the perineal band counter-extension. The dressing above described, was found to effect counter-extension without a particle of pain or soreness.

Since penning that communication, I have received a letter from Dr. P. R. Hoy, of Racine, Wis., who allows me to mention the following improvement, made by him in the above treatment.

On trying the method detailed in the Examiner, one finds two inconveniences. First, the difficulty of raising a patient with a fractured femur so as to apply the straps to the back; and secondly, the softening of the straps on the back by the heat of the body, confined by the bed, so that they gradually slide from their position. Dr. Hoy, therefore, modified the dressing, by cutting the straps full three inches wide and applying them only in front. He also applies three belts, or rather cross straps, which only go half around the body. In this way they are adjusted without moving the patient, and not being heated between the body and the bed, they do not slip. For these reasons, Dr. Hoy claims that his modification of my plan is an improvement, and asserts that in his practice it works admirably. E. Andrews, M. D.

Chicago, Jan., 1862.

---

The practice of salting the streets of New York, after every snow storm, to hasten the melting of the snow, is now forbidden by law. Great injury resulted from it to persons in the street, and also to horses, in consequence of exposure to the intense cold to the feet produced thereby.

